# Supratentorial CNS-PNETs in children; a Swedish population-based study with molecular re-evaluation and long-term follow-up

**DOI:** 10.1186/s13148-023-01456-2

**Published:** 2023-03-09

**Authors:** Elizabeth Schepke, Maja Löfgren, Torsten Pietsch, Teresia Kling, Claes Nordborg, Thomas Olsson Bontell, Stefan Holm, Anders Öberg, Per Nyman, Marie Eliasson-Hofvander, Magnus Sabel, Birgitta Lannering, Helena Carén

**Affiliations:** 1 Childhood Cancer Centre, Queen Silvia Children’s Hospital, Sahlgrenska University Hospital, Gothenburg, Sweden; 2 Sahlgrenska Center for Cancer Research, Department of Medical Biochemistry and Cell Biology, Institute of Biomedicine, Sahlgrenska Academy, University of Gothenburg, Medicinaregatan 1F, 405 30 Gothenburg, Sweden; 3 Department of Neuropathology, DGNN Brain Tumour Reference Centre, University of Bonn Medical Center, Bonn, Germany; 4 Department of Clinical Pathology, Sahlgrenska University Hospital, Gothenburg, Sweden; 5 Departmentof Physiology, Institute of Neuroscience and Physiology, Sahlgrenska Academy, University of Gothenburg, Gothenburg, Sweden; 6 Department of Paediatric Haematology and Oncology, Astrid Lindgrens Childrens Hospital, Karolinska University, Stockholm, Sweden; 7 Department of Woman’s and Children’s Health, Uppsala University, Uppsala, Sweden; 8 Department of Paediatrics, Linköping University, Linköping, Sweden; 9 Department of Paediatric Oncology and Haematology, Lund University, Skåne University Hospital, Lund, Sweden; 10 Department of Clinical Science, Sahlgrenska Academy, University of Gothenburg, Gothenburg, Sweden

**Keywords:** CNS-PNET, Epigenetics, DNA methylation profiling, CNS NB-*FOXR2*, ETMR, Long term survival

## Abstract

**Background:**

Molecular analyses have shown that tumours diagnosed as supratentorial primitive neuro-ectodermal tumours of the central nervous system (CNS-PNETs) in the past represent a heterogenous group of rare childhood tumours including high-grade gliomas (HGG), ependymomas, atypical teratoid/rhabdoid tumours (AT/RT), CNS neuroblastoma with forkhead box R2 (*FOXR2*) activation and embryonal tumour with multi-layered rosettes (ETMR). All these tumour types are rare and long-term clinical follow-up data are sparse. We retrospectively re-evaluated all children (0–18 years old) diagnosed with a CNS-PNET in Sweden during 1984–2015 and collected clinical data.

**Methods:**

In total, 88 supratentorial CNS-PNETs were identified in the Swedish Childhood Cancer Registry and from these formalin-fixed paraffin-embedded tumour material was available for 71 patients. These tumours were histopathologically re-evaluated and, in addition, analysed using genome-wide DNA methylation profiling and classified by the MNP brain tumour classifier.

**Results:**

The most frequent tumour types, after histopathological re-evaluation, were HGG (35%) followed by AT/RT (11%), CNS NB-*FOXR2* (10%) and ETMR (8%). DNA methylation profiling could further divide the tumours into specific subtypes and with a high accuracy classify these rare embryonal tumours. The 5 and 10-year overall survival (OS) for the whole CNS-PNET cohort was 45% ± 12% and 42% ± 12%, respectively. However, the different groups of tumour types identified after re-evaluation displayed very variable survival patterns, with a poor outcome for HGG and ETMR patients with 5-year OS 20% ± 16% and 33% ± 35%, respectively. On the contrary, high PFS and OS was observed for patients with CNS NB-*FOXR2* (5-year 100% for both). Survival rates remained stable even after 15-years of follow-up.

**Conclusions:**

Our findings demonstrate, in a national based setting, the molecular heterogeneity of these tumours and show that DNA methylation profiling of these tumours provides an indispensable tool in distinguishing these rare tumours. Long-term follow-up data confirms previous findings with a favourable outcome for CNS NB-*FOXR2* tumours and poor chances of survival for ETMR and HGG*.*

**Supplementary Information:**

The online version contains supplementary material available at 10.1186/s13148-023-01456-2.

## Introduction

Embryonal tumours of the central nervous system (CNS) in children are a group of malignant, highly aggressive tumours that mostly affect infants and young children, with medulloblastomas accounting for the majority of cases [1–3]. Historically, supratentorial embryonal tumours were often diagnosed as CNS primitive neuroectodermal tumours (CNS-PNETs) but also supratentorial medulloblastomas and cerebral neuroblastomas. The supratentorial CNS-PNETs were mainly located in the cerebral hemispheres and constituted 3–5% of all paediatric brain tumours [4, 5]. These rare tumours often exhibited similar morphology with poorly differentiated small cells and were challenging to classify [6]. Patients diagnosed with CNS-PNETs were mostly treated according to high risk medulloblastoma protocols and the treatment outcomes were poor with 5 -year overall survival (OS) of 41–45% [7–10].

During the last decade molecular markers and epigenetic profiling have revealed that supratentorial CNS-PNETs consists of biologically different tumours [11–13] including tumour types like high-grade gliomas (HGGs), ependymomas (EPNs), atypical teratoid rhabdoid tumours (AT/RTs), embryonal tumours with multi-layered rosettes (ETMRs) as well as other rare embryonal tumours [14]. In a study by Sturm et al., DNA methylation profiles from 323 CNS-PNETs were analysed [12] and four other embryonal tumour types were then defined including CNS neuroblastoma with *FOXR2* activation (CNS NB-*FOXR2*) and CNS high-grade neuroepithelial tumour with *BCOR* alteration (CNS HGNET-*BCOR*). In the latest updated WHO CNS5 classification [15] these two tumour types were incorporated as CNS neuroblastoma, *FOXR2-*activated and CNS tumour with *BCOR* internal tandem duplication (CNS *BCOR* ITD).

In this study, we aimed at re-evaluating tumours diagnosed as supratentorial CNS-PNET in Sweden between 1984 and 2015 and collect clinical data with a very long follow-up time.

## Materials and methods

### Patient population and sample collection

All paediatric patients (< 18 years old) diagnosed with a supratentorial CNS-PNET and registered in the Swedish Childhood Cancer Registry (SCCR) between the 1st of January 1984 and 31st of December 2015 were eligible for the study. We searched the registry for the diagnoses of supratentorial PNET, supratentorial Medulloblastoma or CNS-PNET. The SCCR is estimated to capture approximately 94% of all diagnosed CNS tumours in children (0–15 years old) in Sweden since 1984 (unpublished data).

In total 88 patients were identified in the registry and formalin-fixed paraffin-embedded (FFPE) tumour material was available for re-evaluation in 71 of them. Clinical data were obtained from the SCCR or from the patients´ records. For each patient, gender, age at diagnosis, tumour localisation, metastatic status according to Chang stage [16], date of diagnosis, treatment, date of progression and date of death, or last follow-up were collected. Cases were followed until death, or until the 1st of February 2022.

### Histopathological and molecular re-evaluation

For every tumour case 5–15 unstained sections were collected on glass slides for immunohistochemical analyses and tumour DNA for genetic/molecular analyses. The 71 tumour samples were re-evaluated by an experienced neuropathologist (TP) using state-of-the art diagnostic methods and tumour samples were reclassified according to WHO 2021 [15]. Analyses used for the histopathological re-evaluation are shown in Additional file 1: Table S1.

### DNA methylation analyses

For 63 of the 71 samples, tumour material was sufficient for DNA methylation analyses. DNA was extracted from FFPE tumour tissue and genome-wide DNA methylation profiling was performed using the Infinium Methylation EPIC BeadChip as previously described [17, 18]. Three samples had low DNA quality with a probe failure rate of > 10% and were excluded from further analysis.

Methylation-based classification of the tumours was performed with the Molecular Neuropathology (MNP) brain tumour classifier (https://www.molecularneuropathology.org/mnp/) [19] with its newest and yet unpublished version 12.5. The classifier has four levels for hierarchical clustering: a superfamily, a family, a class and a subclass. A calibrated score (CS) ≥ 0.9 (score range 0–1) was considered a classification match for a methylation family or methylation class/subclass according to the instructions of the classifier [20].

For all samples with a CS < 0.9, the methylation data were visualized with t-distributed stochastic neighbor embedding (t-SNE) with reference profiles from the published MNP data set (Gene Expression Omnibus: GSE90496) [19]. Copy number profiles were estimated from the Infinium Methylation BeadChip data using the *conumee* R package [21].

### Statistical analysis

Statistical analyses were performed in R version 4.2.1 [22]. Progression-free survival (PFS) and OS were calculated according to the Kaplan–Meier method and differences in outcome between patient groups were tested using Log Rank method. PFS was calculated from the date of diagnosis until tumour relapse or last contact for patients who had no signs of disease. OS was calculated from the date of diagnosis until date of death of the patient or to the date 1st of February 2022 for patients who were still alive. The significance level was set to p < 0.05.

## Results

### Patients’ characteristics

Eighty-eight paediatric patients were diagnosed with a supratentorial CNS-PNET in Sweden during 1984–2015. For 71 patients, tumour material for re-evaluation by histopathology was available. There were 39 male and 32 female patients, ratio of 1.2. The median age at diagnosis was 6.2 years (range: 0.2–17.4; mean 7.1). Most tumours arose in the cerebral hemisphere 60/71 (86%) and 11/71 (16%) were located centrally or in the midline. Localised disease was seen in 67 (94%) patients, one patient had M1 at diagnosis and 3 patients M2-3. No patient was staged as Chang M4. In total, 65 children (92%) had a resection and 6 underwent biopsy.

### Histopathological re-evaluation

**Fig. 1:**
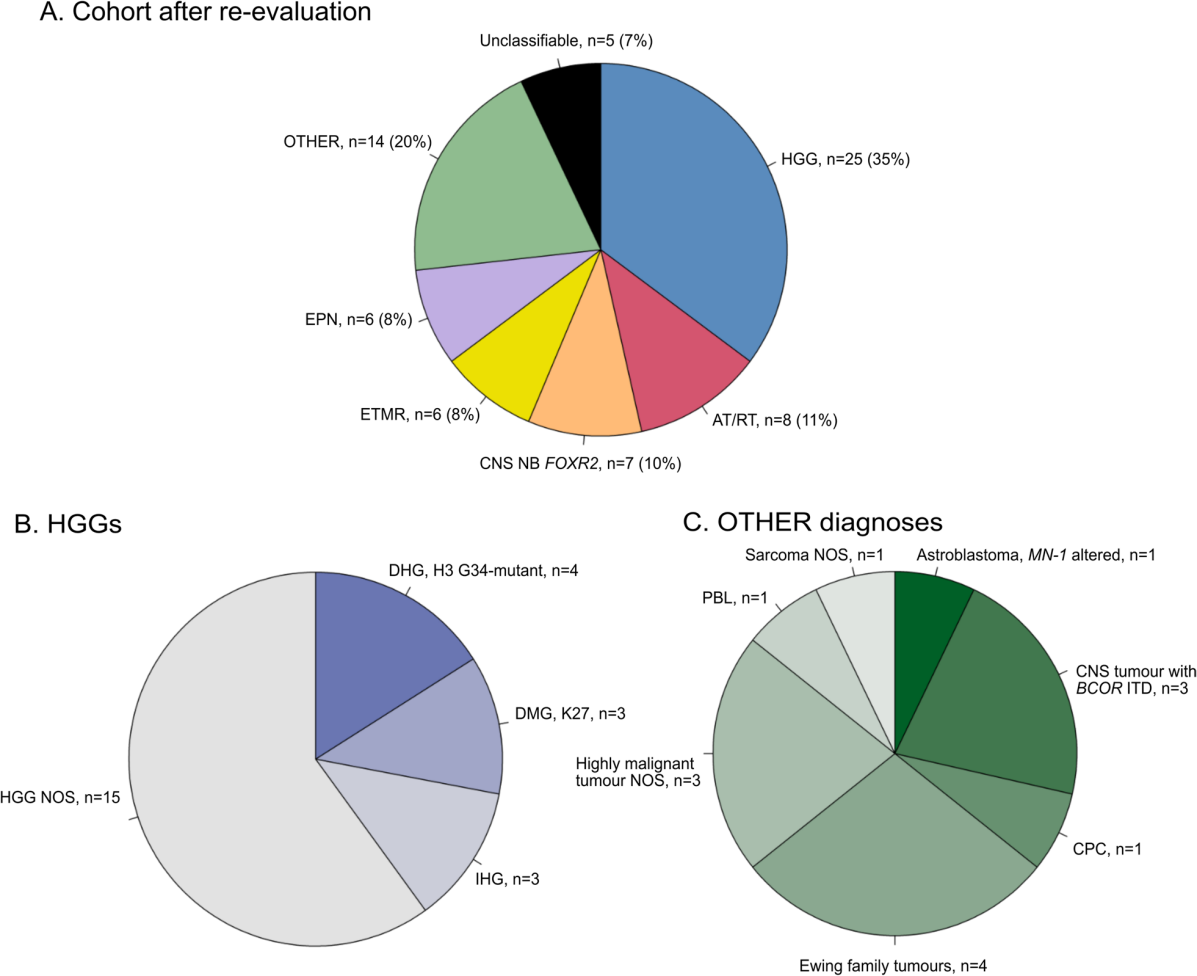
Cohort overview. **A**. Distribution of tumour types after histopathological and molecular re-evaluation of 71 supratentorial CNS-PNETs; **B** and **C**. Further subdivision of the tumour types based on the histopathological re-evaluation; HGGs (*n* = 25) and OTHER diagnoses (*n* = 14) *Abbreviations*: HGG, high-grade glioma; AT/RT, atypical teratoid/rhabdoid tumour; CNS NB-*FOXR2*, CNS neuroblastoma, *FOXR2*-activated; ETMR, embryonal tumour with multilayered rosettes; EPN, ependymoma; DHG, H3 G34-mutant, diffuse hemispheric glioma H3 G34-mutant; DMG, K27, diffuse midline glioma H3 K27-altered; IHG, infant-type hemispheric glioma; HGG NOS, high-grade glioma not otherwise specified; PBL, pineoblastoma; CPC, choroid plexus carcinoma

After histopathological re-evaluation and classification according to the WHO 2021 classification the tumours were divided into the following groups: High-grade glioma (HGG), *n* = 25 (35%), AT/RT, *n* = 8 (11%), CNS NB-*FOXR2*, *n* = 7 (10%), ETMR, *n* = 6 (8%) and ependymoma, *ZFTA*-fusion positive, *n* = 6 (8%) (Fig. 1A). Fourteen cases (20%) were grouped as OTHER and five tumour samples were unclassifiable by neuropathological analysis. Some of the HGGs could be further subclassified as “Diffuse hemispheric glioma, H3 G34-mutant” (*n* = 4), “Diffuse midline glioma, H3 K27-altered” (*n* = 3) and Infant-type hemispheric glioma (*n* = 3) and the rest classified as HGG NOS (*n* = 15) (Fig. 1B). The group OTHER included for example Astroblastoma, *MN-1* altered (*n* = 1), “CNS tumour with *BCOR* ITD” (*n* = 3), “Ewing family tumours” (*n* = 4) and pineoblastoma (*n* = 1) (Fig. 1C). Three tumours were highly malignant (pleomorphic) tumours and could not be further classified.

### DNA methylation-based re-evaluation

**Fig. 2:**
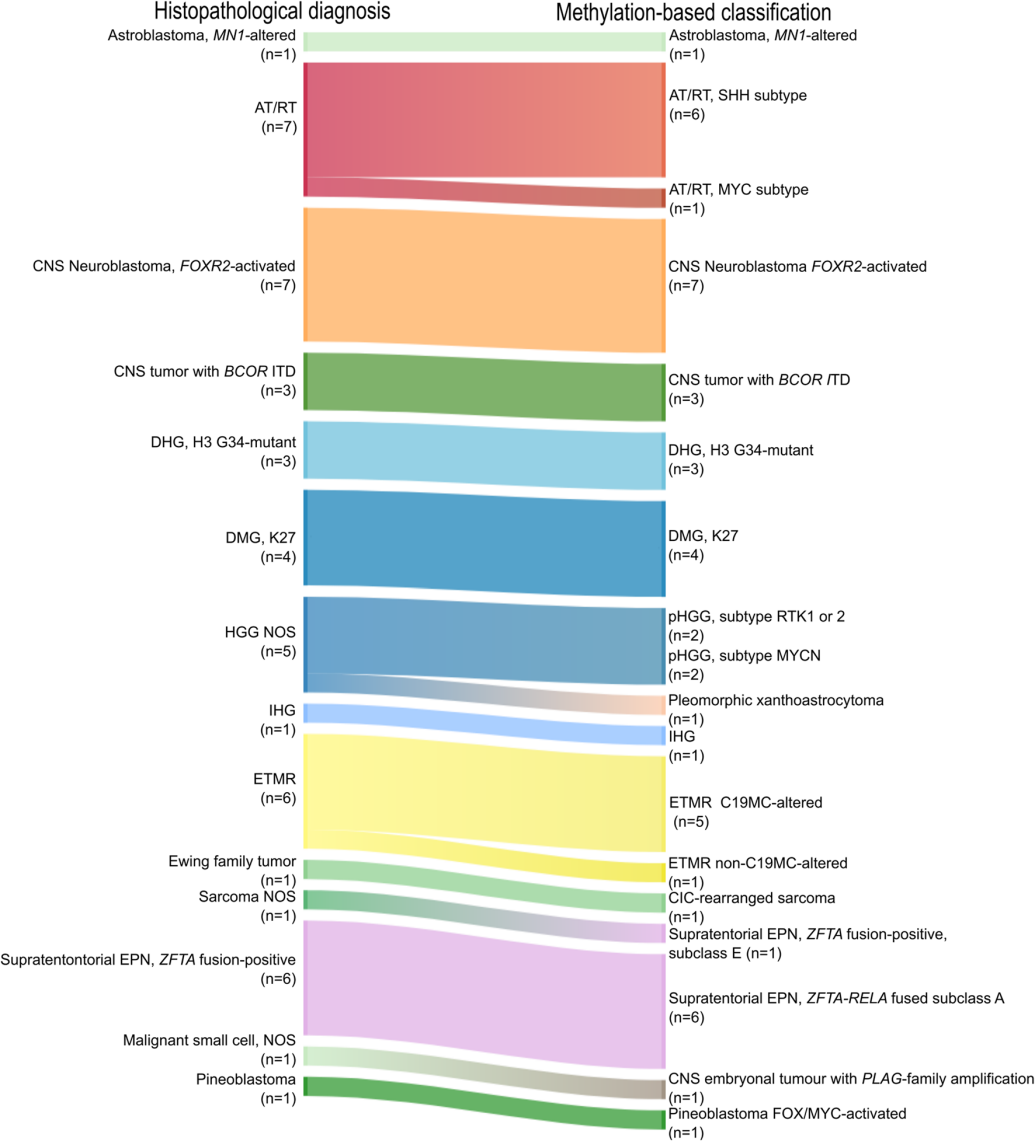
DNA methylation-based classification of 47 CNS-PNETs. Sankey plot of the re-evaluated histopathological diagnoses (WHO 2021) (left) and corresponding methylation group (right). Abbreviations: AT/RT, atypical teratoid/rhabdoid tumour; DHG, H3 G34-mutant, diffuse hemispheric glioma H3 G34-mutant; DMG, K27, diffuse midline glioma H3 K27-altered; HGG NOS, high-grade glioma not otherwise specified; IHG, infant-type hemispheric glioma; ETMR, embryonal tumour with multilayered rosettes

For 60 of 71 (85%) re-evaluated patients, a successful DNA methylation analysis was achieved. In 47 of the 60 samples (78%) a specific methylation group could be assigned with a CS ≥ 0.9 (Fig. 2). In 68% of these cases the DNA methylation-based classification confirmed the histology-based diagnoses. Thus, all CNS NB-*FOXR2s*, CNS tumours with *BCOR* ITDs and ETMRs were well classified including one ETMR without the microRNA cluster on chromosome 19 (*C19MC*). Several of the HGGs and all AT/RTs could be further subdivided into more specific subgroups by methylation array (Fig. 2).

One tumour had the histopathological diagnosis of a sarcoma NOS but was classified by methylation profiling as a supratentorial ependymoma, *ZFTA* fusion-positive, subclass E. The tumour displayed histopathological features of a sarcomatous tumour and showed no nuclear accumulations of p65 (RELA) and might represent an ependymoma with an alternative fusion partner other than *RELA*, as previously described [23]. Another tumour was diagnosed by histopathology as a malignant small cell tumour NOS and by methylation classified as a CNS embryonal tumour with *PLAG*-family amplification. The analysis of copy number alterations (CNAs) showed amplification of the *PLAGL1* gene on chromosome 6 (Additional file 2: Fig. S1).

**Fig. 3:**
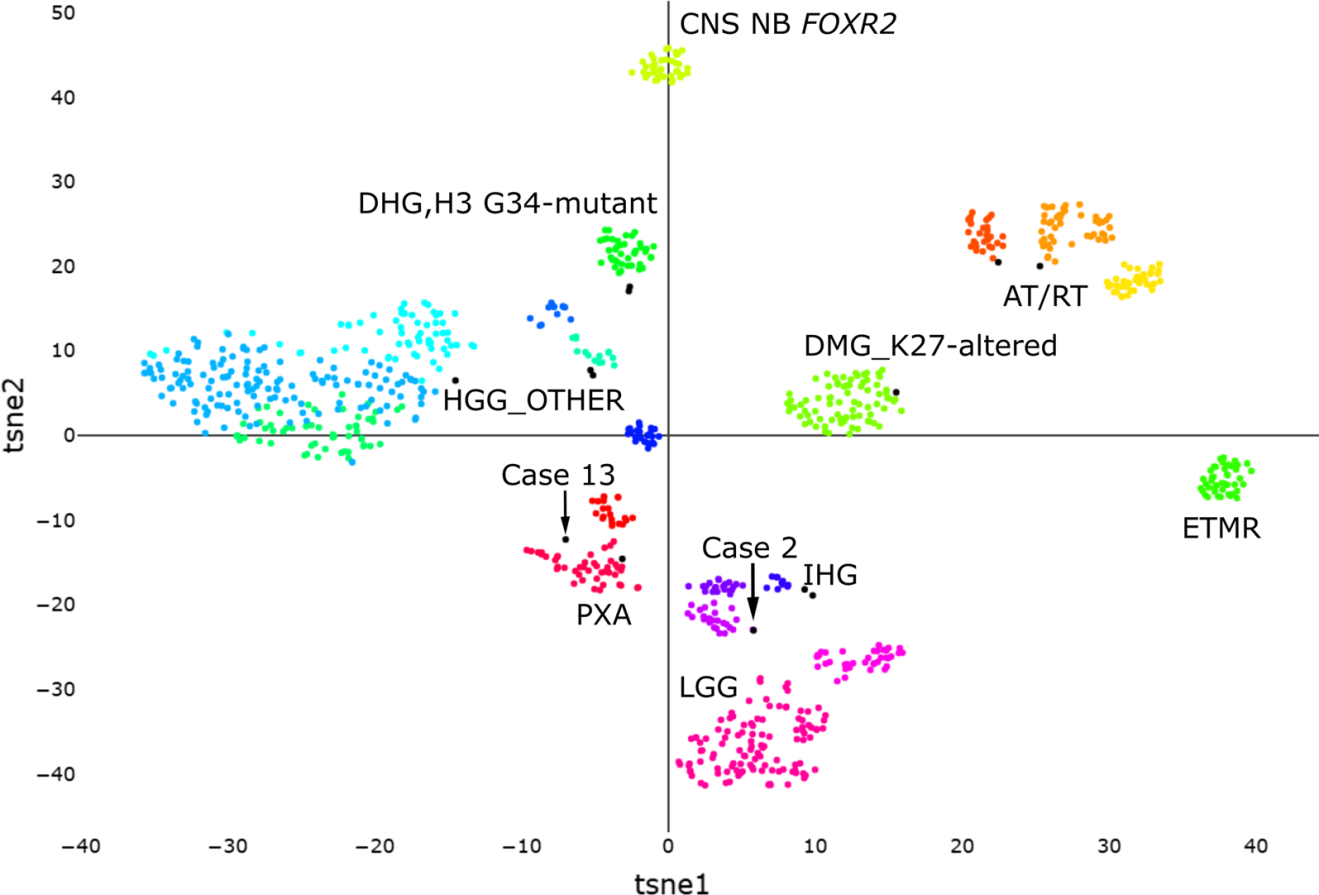
Methylation profiling of 13 CNS-PNETs (black dots) with a calibrated score < 0.9 presented in a t-distributed stochastic neighbor embedding (t-SNE) analysis shows clustering against reference classes from the published MNP data set (only reference samples with diagnoses relevant for this study were included) [19]. Case 2 clustered among the low-grade tumours and case 13 could not be classified by methylation. Abbreviations: AT/RT, atypical teratoid/rhabdoid tumour; CNS NB-*FOXR2*, CNS neuroblastoma *FOXR2*-activated; DMG, K27, diffuse midline glioma H3 K27-altered; ETMR, embryonal tumour with multilayered rosettes; DHG, H3 G34-mutant, diffuse hemispheric glioma H3 G34-mutant; HGG_OTHER, including subtype RTK I, II, mesenchymal and midline; IHG, infant-type hemispheric glioma; PXA, pleomorphic xanthoastrocytoma; LGG, low-grade gliomas

The remaining 13 of 60 tumour samples (22%) could not be confidently matched by methylation-based classification (CS < 0.9) and were visualized in a t-SNE plot (Fig. 3). Most of these tumours clustered close to different subtypes of the reference cohort´s HGGs which was also the diagnosis most of these tumours had been given by the histopathological re-evaluation, listed in Table 1 and Additional file 3: Table S2.

**Table 1:** Histopathological diagnoses and methylation-based classifications of the 13 tumour samples with a calibrated score < 0.9

Cases	Histopathological re-evaluation	Prediction: family	CS: family
1	HGG NOS	Glioblastoma, IDH-wildtype	0.83808
2	HGG NOS	Pilocytic astrocytoma	0.55519
3	HGG NOS	Diffuse pediatric-type high-grade glioma, H3-wildtype and IDH-wildtype	0.82513
4	HGG NOS	Diffuse pediatric-type high-grade glioma, H3-wildtype and IDH-wildtype	0.65948
5	HGG NOS	Glioblastoma, IDH-wildtype	0.28580
6	Diffuse midline glioma, H3-K27M altered	Diffuse midline glioma, H3K27-altered	0.53717
7	Diffuse hemispheric glioma, H3 G34-mutant	Diffuse pediatric-type high-grade glioma, H3-wildtype and IDH-wildtype	0.50395
8	IHG	Diffuse pediatric-type high-grade glioma, H3-wildtype and IDH-wildtype	0.77386
9	Highly malignant tumor NOS	Diffuse pediatric-type high-grade glioma, H3-wildtype and IDH-wildtype	0.50582
10	CPC	Choroid plexus tumours	0.81520
11	AT/RT	Atypical teratoid rhabdoid tumour	0.70174
12	Ewing tumor with CIC::DUX fusion	NA	NA
13	Unclassifiable	Diffuse pediatric-type high-grade glioma, H3-wildtype and IDH-wildtype	0.71402

HGG NOS, High-grade glioma not otherwise specified; IHG, Infant-type hemispheric glioma; AT/RT, atypical teratoid rhabdoid tumour

For one patient (case 2) the DNA methylation classification resulted in a CS of 0.84 for the superfamily low-grade glial/glioneuronal/neuroepithelial tumours (CS of 0.55 for the family Pilocytic astrocytoma) but was by neuropathological re-evaluation diagnosed as HGG NOS. This patient had a partial tumour resection, received focal radiotherapy and chemotherapy, and is a long-term survivor with no evidence of disease 7.5 years after diagnosis which is unusual for a HGG and thus favours the methylation-based diagnosis. Another tumour sample, (case 12), could not be classified by methylation and was by histopathologic classification diagnosed as an Ewing tumour with a CIC::DUX fusion. This could therefore be a tumour type currently not included in the classifier.

### Clinical data and long-term survival

Based on the neuropathological diagnoses the tumours were divided into different tumour groups (Fig. 1A) and clinical data for each group are presented in Table 2.

**Table 2:** Clinicopathological features for patients after re-evaluation

	HGG (*n* = 25)	AT/RT (*n* = 8)	CNS NB-*FOXR2* (*n* = 7)	ETMR (*n* = 6)	EPN (*n* = 6)	OTHER (*n* = 14)
*Age at diagnosis, median (years)*	12.7	2.6	5.3	2.8	5.9	3.3
Gender						
Male	12 (48%)	4 (50%)	3 (43%)	6 (100%)	3 (50%)	9 (64%)
Female	13 (52%)	4 (50%)	4 (57%)	¨	3 (50%)	5 (36%)
*Location of primary tumor*						
Hemisphere	18 (72%)	7 (88%)	7 (100%)	5 (83%)	6 (100%)	9 (64%)
Central/Midline	7 (28%)	1 (12%)	¨	1 (17%)	¨	2 (14%)
Details unknown						3 (22%)
*Metastatic status*						
M0	24 (96%)	7 (88%)	6 (86%)	6 (100%)	6 (100%)	13 (93%)
M1-3	1 (4%)	¨	1 (14%)	¨	¨	1 (7%)
Details unknown		1 (12%)				
*Tumor resection*						
GTR	8 (32%)	3 (38%)	5 (71%)	2 (33%)	4 (67%)	7 (50%)
Partial resection	9 (36%)	1 (12%)	2 (29%)	1 (17%)	1 (17%)	2 (14%)
Biopsy	4 (16%)					
Details unknown	4 (16%)	4 (50%)		3 (50%)	1 (17%)	5 (36%)
*Radiotherapy received*						
Yes	19 (76%)	2 (25%)	7 (100%)	1 (17%)	5 (83%)	7 (50%)
No	6 (20%)	6 (75%)	¨	5 (83%)	1 (17%)	7 (50%)
*Chemotherapy received*						
Yes	21 (84%)	8 (100%)	7 (100%)	5 (83%)	6 (100%)	12 (85%)
No	3 (12%)	¨	¨	1 (17%)	¨	2 (15%)
High-dose chemotherapy with stemcell rescue	¨	1 (12%)	2 (29%)	2 (33%)	¨	2 (14%)
Details unknown	1 (4%)	¨	¨	¨	¨	¨
*Survival data*						
5-year progression free survival	17% ± 17%	25% ± 37%	100%	17% ± 48%	83% ± 21%	36% ± 27%
5-year overall survival	20% ± 16%	25% ± 27%	100%	33% ± 35%	100%	50% ± 26%

HGG, High-grade glioma; AT/RT, atypical teratoid rhabdoid tumour; EPN, ependymoma

For HGG the median age at diagnosis was 12.7 years (range 0.2–17.3). Twelve (48%) were males and 13 females. Tumour location was in the hemispheres for 18 (72%), midline/central in 7 (28%). Twenty-four patients (96%) had a localised disease at diagnosis. Nineteen patients were treated with radiotherapy and of these at least thirteen received craniospinal irradiation (CSI), the type of radiotherapy was not known for the remaining six patients. One patient, three months old at diagnosis, had surgery only and is a long-term survivor. On re-evaluation the diagnose was an Infant-type hemispheric glioma.

**Fig. 4:**
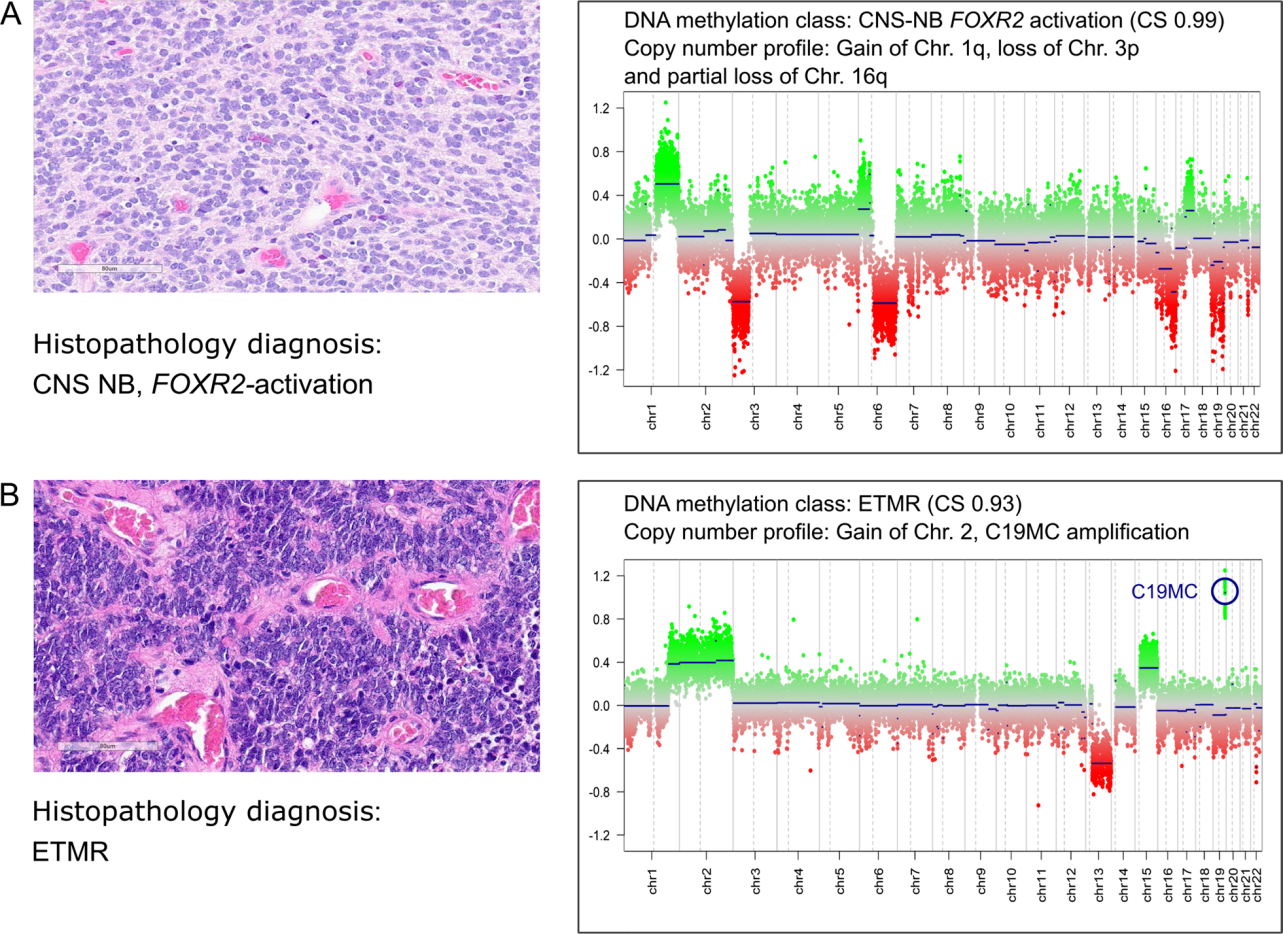
Histopathology (haematoxylin and eosin stain, left) and copy number alterations (CNA, right) plots for **A** CNS NB-*FOXR2-*activated tumour with methylation calibrated score (CS) 0.99 and gain of chromosome 1q, loss of chromosome 3p and partial loss of 16q. **B** Embryonal tumour with multilayered rosettes with methylation CS 0.93 and gain of chromosome 2 and amplification of microRNA cluster on chromosome 19q

Patients with CNS NB-*FOXR2-*activated tumours had a median age at diagnosis of 5.3 years (range 2.5–15.7) and three (43%) were males and four females. All tumours arose in the hemispheres and only one patient had metastatic disease at diagnosis. All patients had received radiotherapy including CSI and two patients had also received high-dose chemotherapy with stem cell rescue (HDSCR). Median CSI dose was 35 Gy (range: 24–36 Gy) and median total tumour dose was 54.6 Gy (range: 49,6–72 Gy). Chromosomal copy number prediction from the DNA-methylation data showed a gain of chromosome 1q in all tumours and loss of whole or partial loss of 16q in 6 out of 7 tumours (86%), as exemplified in Fig. 4A.

All tumours classified as ETMR were diagnosed in young children with the median age of 2.8 years (range 2.1–8.2) and all were males. Five tumours were localised in the hemispheres, and one was centrally located. At diagnosis none were metastatic. One patient died directly after operation, the other five received chemotherapy including HDSCR for two patients who are both long-term survivors. One of the two long-term survivors also received radiotherapy including CSI. On re-evaluation, all the tumours showed the characteristic ependymoblastic rosettes and all were LIN 28 positive by immunohistochemical staining. All tumours except one had the characteristic amplification of *C19MC* and 4/6 tumours had a gain of chromosome 2 (Fig. 4B).

Three patients had the diagnosis of a CNS tumour with *BCOR* ITD both by histopathology and by methylation. The median age was 1.9 years (range 1.8–3.9), two males and one female. All tumours were hemispheric, without metastases. All three patients were treated with chemotherapy. One patient was also treated with HDSCR but no radiotherapy and had a local relapse > 7 years after diagnosis. One patient is a long-term survivor and this patient received radiotherapy including CSI (35 Gy).

The patient with the re-evaluated diagnosis of an Astroblastoma, *MN1-*altered, was a female, and had received chemo- and radiotherapy (incl CSI) and is a long-term survivor (27 years after diagnosis).

**Fig. 5:**
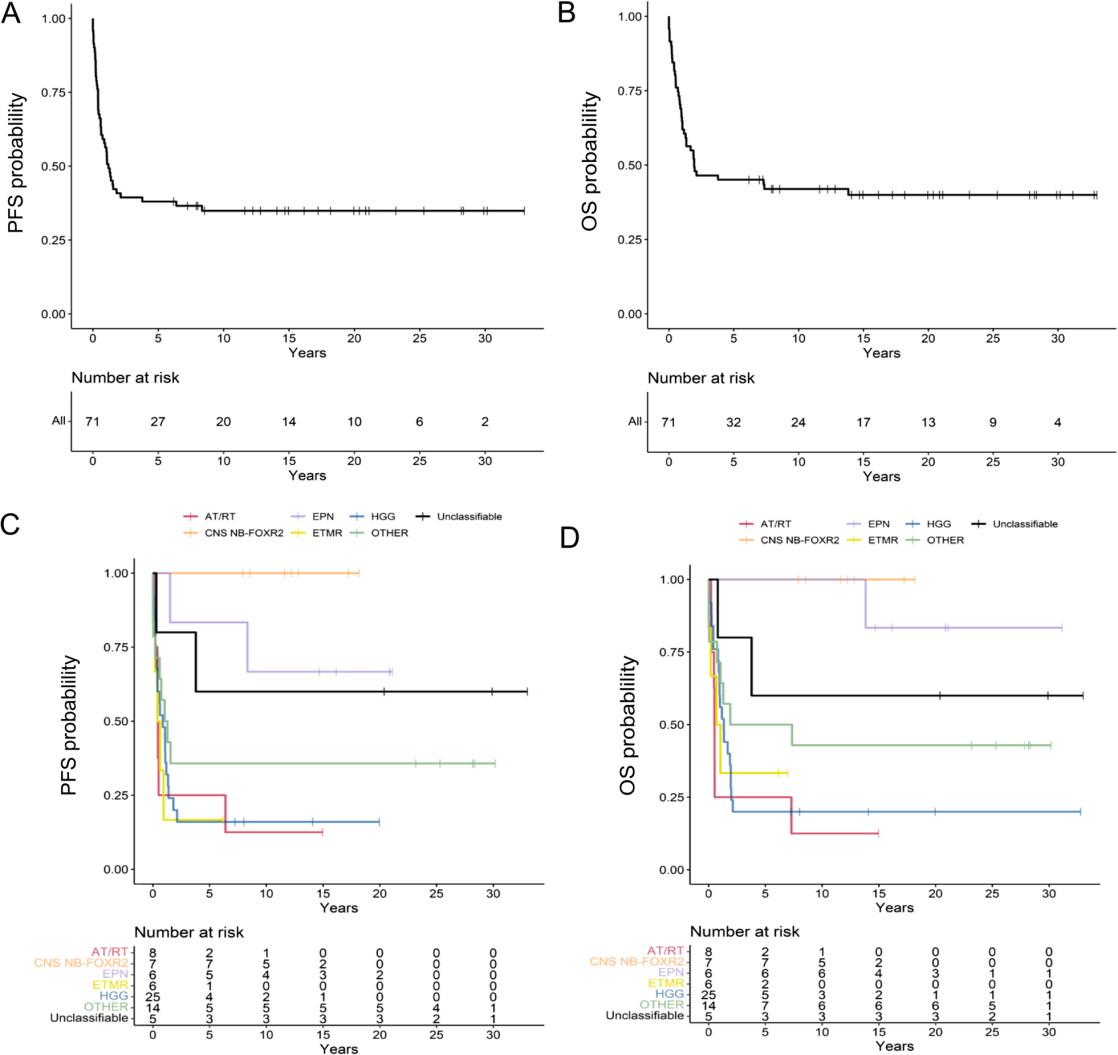
Long-term probability of progression free survival (PFS) and overall survival (OS) of (**A** and **B**) the whole CNS-PNET cohort and (**C** and **D**) for the same cohort of patients grouped according to the histopathological re-evaluation

For the whole CNS-PNET cohort, the mean follow-up time was 8.6 years (range: 0–33.0). The 5 and 10-year PFS for the whole cohort were 38% ± 11% and 35% ± 12%, respectively. The 5-year OS was 45% ± 12% and the 10-year OS was 42% ± 12% (Fig. 5A and B). There was a significant difference in 5-year PFS and OS according to gender with a better PFS and survival for girls compared to boys 50% ± 18% and 28% ± 15% and 56% ± 17% and 36% ± 15%, respectively (Additional file 4: Fig. S2). The survival clearly varied with the re-evaluated tumour types (Fig. 5C and D) with the lowest survival for HGG patients (5-year OS 20% ± 16% which remained the same at 10-year OS) and AT/RT patients (5 and 10-year OS: 25% ± 27% and 12% ± 17%, respectively). Highest survival rates were observed for CNS NB-*FOXR2* patients with 5-year PFS and OS of 100%, respectively which did not change even after 15-years of follow up (Fig. 5C and D). Low survival rates were seen for ETMR patients, 5-year PFS and OS were 17% ± 48% and 33% ± 35%, respectively (Fig. 5C and D). The patient with malignant small cell tumour NOS and *PLAG*-family amplification had received chemo- and radiotherapy in one form (focal or CSI not known) and is a long-term survivor (26 years of follow up).

## Discussion

In this study we retrospectively analysed a series of 71 poorly differentiated supratentorial malignant tumours in children, previously diagnosed as CNS-PNETs. During the last decade it has become evident that this group of tumours consists of a heterogenous mixture of different molecular entities with different clinical behaviour. Our national based tumour-cohort further supports this.

All tumours were re-evaluated by a highly experienced neuropathologist who made state-of-the-art analyses. The resulting distribution of tumour types was in line with previous publications [8, 12, 24]. The majority of the re-evaluated CNS-PNETs could be classified as of different types of HGGs, followed by AT/RTs and CNS NB-*FOXR2* and a minor part consisted of ETMR and other rare CNS embryonal tumours.

For 85% of the tumours, sufficient tumour material was available for further analysis with genome-wide DNA-methylation profiling. In 78%, the histopathological diagnoses for these challenging tumours could be confirmed and/or further subclassified to a more precise subtype. One of the benefits from methylation-based classification is that it is user-independent, whereas inter- and intra-observer variability have been reported for neuropathological diagnostics [25, 26]. We should keep in mind that the re-evaluation of this cohort was performed by a very experienced reference neuropathologist and the importance of using DNA methylation analyses for theses rare embryonal tumours are of even greater help in routine diagnostics.

As this study was retrospective, the tumour material had been archived since many years, sometimes sparse and of low quality. Despite that, DNA-methylation profiling could accurately classify many of the difficult-to-diagnose cases including all CNS NB-*FOXR2*-activated tumours, all ETMRs and all CNS tumours with *BCOR* ITD. It is thus an important tool in the diagnostics of rare embryonal tumours.

One of the CNS-PNET tumours, from a long-term survivor, was by methylation classified as a CNS embryonal tumour with *PLAG*-family amplification, a proposed novel rare paediatric CNS tumour type characterized by amplification of one of the PLAG family genes (*PLAGL1* or *PLAGL2*) and a specific methylation profile [27]. This tumour is not included in the current WHO CNS5 classification [15] and is scarcely reported in the literature. More clinicopathological characteristics of this rare tumour is anticipated.

Thirteen of the methylation profiled tumours in our cohort (22%) could not be confidently classified by the current methylation classifier version and there are several reasons why a calibrated score can be lower (CS < 0.9). [28]. Factors such as poor DNA quality, lower tumour cell content or that the tumour type itself is not represented in the classifier affects the classification [17, 28, 29]. A calibrated score < 0.9 can still give valuable information and when the thirteen tumours were visualized in a t-SNE analysis we saw that several of the samples clustered around the HGG clusters reflecting molecular similarities to these tumours which was in consistence with the re-evaluated neuropathological diagnoses. Some tumours did not cluster to any known tumour type visualizing the rarity of these tumours. Two previous studies [12, 30], that focused on tumours historically diagnosed as CNS-PNETs and diagnostically challenging cases, reported that approximately 15% of the tumours in these cohorts were likely new entities and indeed it seems that, as in our cohort, there is a group of CNS tumours that eventually will be further characterised both pathologically and clinically.

Overall, all of these newly described embryonal tumour types are rare and specific clinical and treatment data are sparse. They are a major challenge in both diagnostics and clinical management and long-term clinical follow-up data are lacking. During the last years, retrospective studies have revealed that overall survival for ETMR is very poor despite intensive treatment [24, 31–33]. This tumour often progresses early despite therapy which also was shown in this cohort. There were two long term survivors, both had received HDSCR and one also CSI. This sample size is too small to draw any further conclusions. Overall, this data supports the need for novel therapeutic options for these patients.

Two recent studies have observed that treatment including CSI seems to be important for survival of patients with CNS NB-*FOXR2-*activated tumours [24, 34]. All patients diagnosed with a CNS NB-*FOXR2*-tumour in our cohort are long-term survivors. As the numbers are small, only seven patients, firm conclusions cannot be drawn regarding treatment, but our data support the suggested importance of craniospinal radiation for survival [24]. However randomised studies are needed for confirmation on the other hand this will be difficult to achieve due to the rarity of these tumours. Retrospective studies like ours, can hopefully increase the molecular and clinical knowledge on these rare tumours which can help to find new possible therapeutic treatment options.

Treatment data and long-term follow up data was available for all the 71 patients in the cohort. When studied as a whole cohort, 5-years OS was poor (45% ± 12%) decreasing to 42% ± 12% after 10 years which is in line with other publications [7–10]. Surprisingly, there was a significant better outcome for females compared to males which has not been observed in other studies [7, 8]. This is most probably due to that all six patients with an ETMR tumour were boys. However, after dividing the patients according to the re-evaluated diagnoses, we noted that survival varied extensively between different tumour groups. Since more than half of the tumour samples consisted of HGGs, AT/RTs and ETMRs i.e., tumour groups with the poorest survival, these diagnoses contributed to the low survival of the entire group.

## Conclusions

In conclusion, our findings demonstrate the molecular heterogeneity of paediatric cerebral neoplasms previously diagnosed as supratentorial CNS-PNET. Long-term follow-up data confirms previous findings with a favourable outcome for CNS NB-*FOXR2* tumours and poor chances of survival for ETMR and HGG*.* DNA-methylation profiling provides an indispensable tool in distinguishing these rare tumours and should therefore be implemented in initial neuropathological diagnostics.

## Supplementary Information

**Additional file 1.** State of the art diagnostic methods for selected tumor types previously diagnosed as CNS-PNET.

**Additional file 2**: **Figure S1**: CNA plot of the methylation-based diagnosis of CNS embryonal tumour with PLAG-family amplification with methylation calibrated score (CS) 0.99 showing amplification of the *PLAGL1* gene on (A) chromosome 6 and (B) specific gene amplification.

**Additional file 3.** Histopathological diagnoses and corresponding methylation class for the 13 tumour samples with a calibrated score < 0.9.

**Additional file 4**: **Figure S2**: Long-term probability of progression-free survival (PFS) and overall survival (OS) of the whole CNS-PNET cohort, females (red) compared to males (turquoise).

## Data Availability

The datasets generated during the current study are available from the corresponding author on reasonable request.

## Abbreviations

AT/RT: Atypical teratoid rhabdoid tumour
*BCOR*: Bcl6 corepressor
CNA: Copy number alteration
CNS-PNET: CNS primitive neuroectodermal tumour
CSI: Craniospinal irradiation
EPN: Ependymoma
ETMR: Embryonal tumours with multi-layered rosettes
FFPE: Formalin-fixed paraffin-embedded
*FOXR2*: Forkhead box R2
HGG: High-grade glioma
MNP: Molecular neuropathology
OS: Overall survival
PFS: Progression-free survival
PNET: Primitive neuroectodermal tumour
*PLAG*: Pleomorphic adenoma gene
*PLAGL*: Pleomorphic adenoma gene-like
*ZFTA*: Zinc finger translocation associated
