# Supplementary material for: Supratentorial CNS-PNETs in children; a Swedish population-based study with molecular re-evaluation and long-term follow-up
Source: Clin Epigenetics. 2023 Mar 9;15:40. doi: 10.1186/s13148-023-01456-2 (PMC9996973; doi:10.1186/s13148-023-01456-2)
Supplement: Supplementary file 1 — Additional file 1. State of the art diagnostic methods for selected tumor types previously diagnosed as CNS-PNET. [file 13148_2023_1456_MOESM1_ESM.pdf]

Supplementary table 1. State of the art diagnostic methods for selected tumor types previously diagnosed as CNS PNET

**Tumor diagnosis**	**Immunohistochemistry markers**	**Genetic techniques**	**Other molecular techniques**
Astroblastoma, *MN1* -altered	GFAP positive<br>EMA positive<br>OLIG2 at least focally positive	FISH with break-apart *MN1* probe to detect fusion	
Atypical teratoid/rhabdoid tumor	EMA positive<br>Vimentin positive<br>SMA and cytokeratin frequently positive<br>Nuclear loss of Ini-1 or Brg1 protein	*SMARCB1* or *SMARCA4* mutations can be identified by DNA sequencing.<br>Larger deletions can be detected by FISH with specific probes or array-based methods (CGH, MIP)	
Choroid Plexus Carcinoma	Cytokeratin positive<br>EMA positive<br>p53 nuclear accumulation in TP53 mutant cases	Detection of chromosomal losses by array-based methods (CGH, MIP)	
CNS neuroblastoma, *FOXR2* -activated	OLIG2 positive<br>Synaptophysin positive<br>TTF-1 frequently positive<br>GFAP negative<br>Vimentin negative	Almost all cases show gain of chromosome 1q, loss of 16q is also frequent.<br>Detection by FISH with specific probes or array-based methods (CGH, MIP)	Detection of mRNA overexpression of *FOXR2* and *NKX2.1* by quantitative RT-PCR or Nanostring technology
CNS tumor with *BCOR* internal tandem duplication	Vimentin positive<br>CD56 (NCAM) often positive<br>*BCOR* nuclear positive	Internal tandem duplication of *BCOR* detected by PCR	
Embryonal tumor with multilayered rosettes	LIN 28 positive<br>Vimentin positive<br>Synaptophysin positive in neuropil-like matrix	*C19MC* amplification detected by FISH or array-based methods (CGH, MIP)<br>For *C19MC* non-amplified cases, DNA sequencing for *DICER1* mutations	
Ependymoma *ZFTA* fusion-positive	EMA positive<br>Vimentin positive<br>Nuclear accumulation of p65-RelA in most cases	FISH with break-apart *ZFTA* probe<br>RT-PCR or Nanostring technology to detect *ZFTA* fusion transcripts	
HGG (different types)	GFAP positive<br>OLIG2 positive (all but H3-G34 mutant HGG)<br>Vimentin positive<br>H3-K27me3 negative and H3K27M mostly positive and in diffuse midline gliomas<br>H3G34R/V positive in hemispheric gliomas with H3-G34 mutation<br>BRAFV600E often positive in anaplastic PXA /epithelioid GBM	DNA (Pyro)sequencing for mutations of H3F3A, other Histone genes, IDH1, IDH2, BRAF (V600)<br>FISH with break-apart probes to detect fusions of *NTRK1, -2, -3, ALK, ROS, MET* in Infantile hemispheric gliomas. RNA-based methods to detect fusion transcripts<br>(RT-PCR, Nanostring technology, RNA-Seq)	
Pineoblastoma	OT2 and OTX 3 positive<br>Synaptophysin positive<br>Vimentin negative		

GFAP, glial fibrillary acidic protein; EMA, epithelial membrane antigen; OLIG2, oligodendrocyte transcription factor 2; SMA, smooth muscle actin; TTF-1, Thyroid transcription factor-1; HGG, high grade glioma
