# Supplementary figures and images for: Supratentorial CNS-PNETs in children; a Swedish population-based study with molecular re-evaluation and long-term follow-up
Source: Clin Epigenetics. 2023 Mar 9;15:40. doi: 10.1186/s13148-023-01456-2 (PMC9996973; doi:10.1186/s13148-023-01456-2)

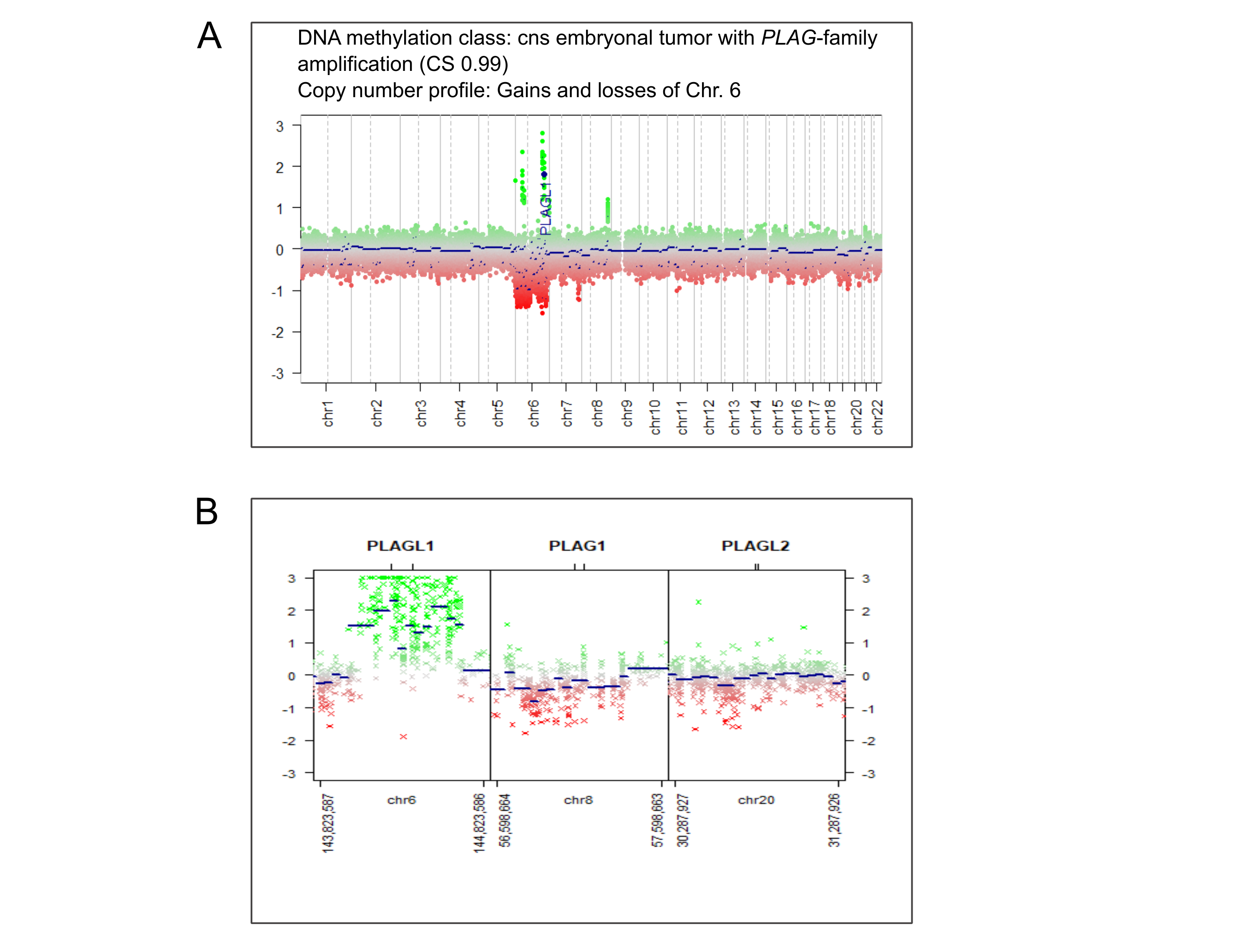

Supplement: Supplementary file 2 — Additional file 2: Figure S1: CNA plot of the methylation-based diagnosis of CNS embryonal tumour with PLAG-family amplification with methylation calibrated score (CS) 0.99 showing amplification of the PLAGL1 gene on (A) chromosome 6 and (B) specific gene amplification. [file 13148_2023_1456_MOESM2_ESM.tif]

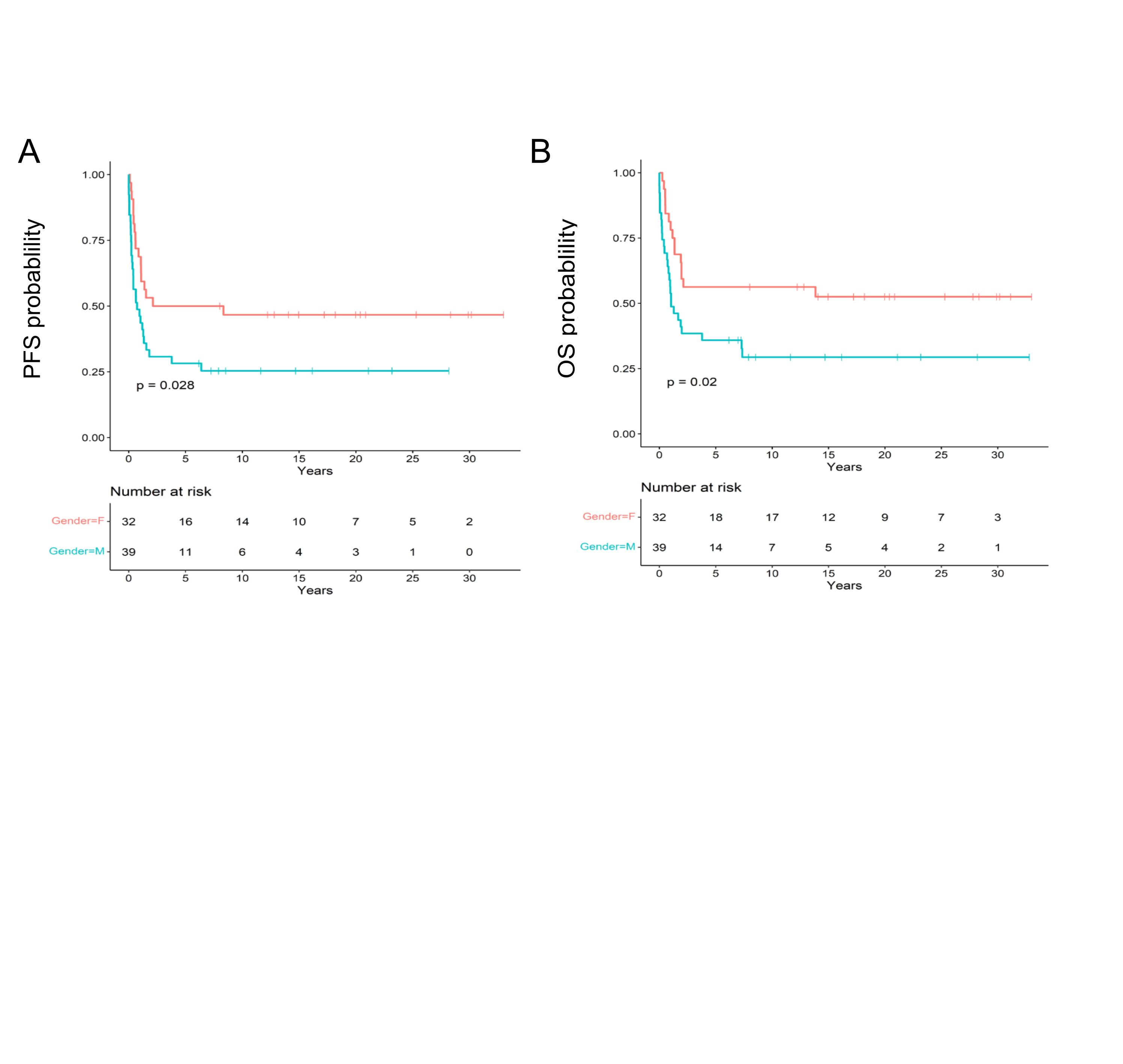

Supplement: Supplementary file 4 — Additional file 4: Figure S2: Long-term probability of progression-free survival (PFS) and overall survival (OS) of the whole CNS-PNET cohort, females (red) compared to males (turquoise). [file 13148_2023_1456_MOESM4_ESM.tiff]
