# Supplementary material for: Supratentorial CNS-PNETs in children; a Swedish population-based study with molecular re-evaluation and long-term follow-up
Source: Clin Epigenetics. 2023 Mar 9;15:40. doi: 10.1186/s13148-023-01456-2 (PMC9996973; doi:10.1186/s13148-023-01456-2)
Supplement: Supplementary file 3 — Additional file 3. Histopathological diagnoses and corresponding methylation class for the 13 tumour samples with a calibrated score < 0.9. [file 13148_2023_1456_MOESM3_ESM.pdf]

Supplementary table 2.

Case ID	Histopathological re-evaluation	Superfamily		Family		Class		Subclass	
		Prediction	CS	Prediction	CS	Prediction	CS	Prediction	CS
1	HGG NOS	Adult-type diffuse gliomas	0.85	Glioblastoma, IDH-wildtype	0.84	Glioblastoma, IDH-wildtype, mesenchymal type	0.83	Glioblastoma, IDH-wildtype, mesenchymal subtype	0.60
2	HGG NOS	Low-grade glial/glioneuronal/neuroepithelial tumours	0.84	Pilocytic astrocytoma	0.56	Supratentorial midline pilocytic astrocytoma	0.46	Pilocytic astrocytoma, midline	0.46
3	HGG NOS	Paediatric-type diffuse high-grade gliomas	0.84	Diffuse pediatric-type high-grade glioma, H3-wildtype and IDH-wildtype	0.83	Diffuse paediatric-type high grade glioma, rtk2 subtype	0.59	Diffuse paediatric-type high grade glioma, rtk2 subtype, subclass b (novel)	0.57
4	HGG NOS	Paediatric-type diffuse high-grade gliomas	0.66	Diffuse pediatric-type high-grade glioma, H3-wildtype and IDH-wildtype	0.66	Diffuse paediatric-type high grade glioma, rtk1 subtype	0.64	Diffuse paediatric-type high grade glioma, rtk1 subtype, subclass a (novel)	0.50
5	HGG NOS	Adult-type diffuse gliomas	0.32	Glioblastoma, IDH-wildtype	0.29	High-grade diffuse glioma of the midline/posterior fossa;H3/IDH-wildtype	0.21	Glioblastoma, IDH-wildtype, subtype posterior fossa (novel)	0.21
6	Diffuse midline glioma, H3-K27M altered	Paediatric-type diffuse high-grade gliomas	0.61	Diffuse midline glioma, H3K27-altered	0.54	Diffuse midline glioma, H3 K27-mutant/ EZHIP overexpressing	0.53	Diffuse midline glioma, H3 K27-altered, subtype H3 K27-mutant or EZHIP expressing	0.53
7	Diffuse hemispheric glioma, H3 G34-mutant	Paediatric-type diffuse high-grade gliomas	0.51	Diffuse pediatric-type high-grade glioma, H3-wildtype and IDH-wildtype	0.50	Diffuse hemispheric glioma, H3 G34-mutant	0.44	Diffuse hemispheric glioma, H3 G34-mutant	0.44
8	IHG	Paediatric-type diffuse high-grade gliomas	0.82	Diffuse pediatric-type high-grade glioma, H3-wildtype and IDH-wildtype	0.77	Diffuse paediatric-type high grade glioma, rtk1 subtype	0.76	Diffuse paediatric-type high grade glioma, rtk1 subtype, subclass a (novel)	0.49
9	Highly malignant tumor NOS	Paediatric-type diffuse high-grade gliomas	0.53	Diffuse pediatric-type high-grade glioma, H3-wildtype and IDH-wildtype	0.51	Diffuse paediatric-type high grade glioma, H3 wildtype and IDH wild type, Subtype A&B (novel)	0.17	Diffuse paediatric-type high grade glioma, H3 wildtype and IDH wild type, Subtype A (novel)	0.17
10	CPC	Choroid plexus tumours	0.82	Choroid plexus tumours	0.82	Choroid plexus carcinoma	0.81	Choroid plexus carcinoma, adult subtype (novel)	0.81
11	AT/RT	Other CNS embryonal tumours	0.73	Atypical teratoid rhabdoid tumour	0.70	Atypical teratoid rhabdoid tumour, SHH activated	0.60	Atypical teratoid/rhabdoid tumour, SHH-subtype	0.60
12	Ewing tumor with CIC::DUX fusion	NA	NA	NA	NA	NA	NA	Glioblastoma, idh-wildtype, mesenchymal subtype, subclass b (novel)	0.10
13	Unclassifiable	Paediatric-type diffuse high-grade gliomas	0.72	Diffuse pediatric-type high-grade glioma, H3-wildtype and idh-wildtype	0.71	Diffuse paediatric-type high grade glioma, mycn subtype	0.67	Diffuse paediatric-type high grade glioma, mycn subtype	0.67
